# Diversity of Endophytes of *Actinidia arguta* in Different Seasons

**DOI:** 10.3390/life14010149

**Published:** 2024-01-19

**Authors:** Yingxue Liu, Wenpeng Lu, Yang Li, Boyu Zhai, Baoxiang Zhang, Hongyan Qin, Peilei Xu, Yiming Yang, Shutian Fan, Yue Wang, Changyu Li, Jianjun Zhao, Jun Ai

**Affiliations:** 1 Institute of Special Animal and Plant Sciences, Chinese Academy of Agricultural Sciences, Changchun 130112, China; liuyingxue@caas.cn (Y.L.); luwenpeng@caas.cn (W.L.); zbx0319@126.com (B.Z.); qinyan11@163.com (H.Q.); xupeilei@caas.cn (P.X.); yym0312@163.com (Y.Y.); fanshutian@caas.cn (S.F.); y1989w@126.com (Y.W.); lichangyu@caas.cn (C.L.); 2 College of Animal Science and Veterinary Medicine, Heilongjiang Bayi Agricultural University, Daqing 163319, China; ly18646589171@163.com (Y.L.); zby19940526@byau.edu.cn (B.Z.); zhaojianjun@byau.edu.cn (J.Z.); 3 College of Horticulture, Jilin Agricultural University, Changchun 130112, China

**Keywords:** Illumina MiSeq, *Actinidia arguta*, endophytic bacteria, 16S rRNA, ITS rDNA, different seasons

## Abstract

The seasonal changes in environmental conditions can alter the growth states of host plants, thereby affecting the living environment of endophytes and forming different endophytic communities. This study employs Illumina MiSeq next-generation sequencing to analyze the 16SrRNA and ITS rDNA of endophytes in 24 samples of *Actinidia arguta* stem tissues across different seasons. The results revealed a high richness and diversity of endophytes in *Actinidia arguta*, with significant seasonal variations in microbial community richness. This study identified 897 genera across 36 phyla for bacteria and 251 genera across 8 phyla for fungi. Notably, 69 bacterial genera and 19 fungal genera significantly contributed to the differences in community structure across seasons. A distinctive feature of coexistence in the endophytic community, both specific and conservative across different seasons, was observed. The bacterial community in winter demonstrated significantly higher richness and diversity compared to the other seasons. Environmental factors likely influence the optimal timing for endophyte colonization. Solar radiation, temperature, precipitation, and relative humidity significantly impact the diversity of endophytic bacteria and fungi. In addition, seasonal variations show significant differences in the nutritional modes of fungal endophytes and the degradation, ligninolysis, and ureolysis functions of bacterial endophytes. This study elucidates the potential role of endophytes in assisting *Actinidia arguta* in adapting to seasonal changes and provides a theoretical basis for further exploration of functional microbial strains.

## 1. Introduction

Endophytes are microorganisms that reside in plant tissues without harming the host and are present in almost every plant species [1]. Most endophytes beneficially influence plant growth, and without them plants would struggle to combat biotic and abiotic stresses [2]. Endophytes play critical roles in plant ecology, evolution, and development, including in nitrogen fixation [3], growth promotion [4], and the production of antimicrobial and antitumor substances [5]. They are also pivotal in enhancing plant stress resistance [6] and in environmental pollution remediation [7].

Recent studies confirmed the crucial role of various plant endophytes in helping hosts adapt to biotic stresses and adverse environmental conditions [8]. Wang et al. isolated three species of endophytic fungi, *Trichoderma* spp., from the roots of Gentiana, which, when inoculated, increased the susceptibility of Gentiana to leaf spot disease [9]. Adhikari and Pandey isolated endophytic bacteria *Burkholderia GBPI_TWL* and *Enterobacter GBPI_TWr* from the roots of Taxus wallichiana Zucc. Both bacteria could grow at a wide range of temperatures (5–40 °C, opt = 25 °C) and pHs (1.5–11.0, opt = 6–7), and could tolerate salt concentrations up to 12% [10]. Li et al. used the cold-sensitive banana variety Musa acuminate as the material to study the impact of the endophytic fungus *Piriformospora indica* on the cold resistance of bananas. It was concluded that *Piriformospora indica* confers bananas with enhanced cold resistance by stimulating antioxidant capacity, SS accumulation, and the expression of cold-responsive genes in leaves [11]. Jaiswal and others isolated the endophytic bacteria Enterobacter cloacae subsp. cloacae strain CPR5B and the endophytic fungus *Penicillium citrinum* strain *CPL1F* from the drought-resistant plant Calotropis procera. Both were found to produce indole acetic acid (IAA) and 1-aminocyclopropane-1-carboxylic acid (ACC) deaminase, and were capable of solubilizing phosphate, thus promoting plant growth [12]. Zhang et al. isolated the endophytic fungus *Aspergillus ochraceus XZC-1* from Lagopsis supina, which showed significant antibacterial activity against *Staphylococcus* and *Fusarium graminearum*. The secondary metabolites produced by this fungus, particularly 2-methoxy-6-methyl-1,4-benzoquinone and penicillic acid, were found to exhibit selective cytotoxicity towards cancer cells [13]. Endophytes assist plants in coping with abiotic stress while also being constrained by environmental factors such as intense UV radiation, drought and extreme cold, which can limit the vitality of some endophytes [14]. Hence, environmental conditions not only affect plant species and growth status but also define microbial community structure. In extreme environments, through dual selection by plants and the environment, specialized endophyte resources can become enriched, forming an adaptive capability to adverse conditions within plant tissues [15].

Many studies have shown that seasonal changes are determining factors in the composition of endophytic microbial communities in plants. For instance, different seasons dictate the composition of the endophytic microbial communities in *Kalidium schrenkianum*. The highest diversity of endophytic bacteria was observed during summer and autumn, while the diversity of endophytic fungal communities peaked in spring [16]. The endophytic bacteria in the bark and leaves of *Eucommia ulmoides* exhibit pronounced seasonal and tissue-specific characteristics. The seasonal characteristics are associated with the accumulation of active components of *Delftia* and *Sphingomonas paucimobilis* in medicinal tissues [17]. The diversity of endophytic fungi in the roots, stems, and leaves of *Huperzia serrata* varies significantly across different seasons, with lower diversity observed during summer compared to other seasons [18]. Furthermore, research indicates that the season is a key factor in determining the endophytic bacterial community in mulberry trees, followed by host varieties [19].

*Actinidia arguta*, a large deciduous vine in the *Actinidia* genus of the Actinidiaceae family, is a valuable cold-resistant fruit tree native to China. Its peach-like fruit is uniquely flavored and rich in proteins, amino acids, dietary fibers, and active ingredients like polyphenols, volatile oils, and proanthocyanidins [20] offering anti-cancer, antioxidant and laxative properties [21]. The plant has a well-developed root system and vigorous growth, living up to a century. It withstands temperatures as low as −40 °C [22], making it the most cold-resistant species in the *Actinidia* genus. Compared to *Actinidia chinensis* and *Actinidia deliciosa*, it exhibits greater resistance to ulcer disease [23]. Based on the plant characteristics of *Actinidia arguta*, we hypothesize the presence of microbial strains within *Actinidia arguta* that exhibit growth-promoting, disease-resistant, and cold-tolerant functionalities. By employing high-throughput sequencing methods, we aim to delineate the community structure of endophytic microbes residing within *Actinidia arguta*. This effort will serve as a foundational step towards further exploration and identification of microbial resources with functional properties within *Actinidia arguta*.

Traditional cultivation methods can only recover 0.1–10% of beneficial plant endophytes [24]. In recent years, the development of the Illumina MiSeq second-generation gene sequencing technology has not only allowed for in situ analysis of microbial population structures in their natural state, providing a comprehensive and objective determination of microbial community composition in target environments [25], but has also assisted in studying the diversity of symbiotic microbial ecological functions [26]. To explore the community structure and dynamic changes of endophytes in *Actinidia arguta*, as well as the relationships between endophytes and environmental factors and their functions, this study employed Illumina MiSeq sequencing technology to analyze the endophytic community structure and diversity in *Actinidia arguta* across different seasons. The results will guide our understanding of the microecosystem of *Actinidia arguta* and aid in the exploration of potential functional microbes in disease prevention.

## 2. Materials and Methods

### 2.1. Sample Collection and Processing

This study was conducted from January to December 2021, with samples collected from the *Actinidia arguta* orchard of the Institute of Special Animal and Plant Sciences, Chinese Academy of Agricultural Sciences, Changchun of Jilin province (N 44°00′; E 126°01′). Healthy, vigorously growing plants, free from diseases and pests, were chosen for sampling. Due to the low temperatures in winter, the plants had shed their leaves and the soil was frozen, making it impossible to collect leaf and root samples. Therefore, we selected biennial stem tissues in spring, summer, autumn, and winter as the study materials (Table 1). In each season, stems were collected from 18 plants, with 3 plant stems combined to create one sample, totaling 24 samples overall. After collection, the stems were temporarily stored in a foam box with ice packs. They were transported to the laboratory within an approximately one-hour drive and were processed immediately. Stems were rinsed under running water for 30 min and cut into 2–3 cm segments in a laminar flow hood (ZHJH-C1112C, Shanghai, China). The segments were first disinfected with 75% ethanol for 90 s, washed 3 times with sterile water and then continuously shaken in 2.5% sodium hypochlorite (NaOCl) for 8 min. Finally, sterilized stems were washed 3 times with sterile water and excess water was removed using sterile filter paper [27]. The sterile material was cut into 0.2 cm thick stem segments and stored in new sterile tubes at −80 °C in a cryogenic refrigerator (700 Series, Thermo, Waltham, MA, USA) for total endophytic DNA extraction. All samples were sequenced in a single batch to avoid machine-related errors.

### 2.2. Genomic DNA Extraction, PCR Amplification, and Sequencing

Total microbial genomic DNA was extracted from *Actinidia arguta* samples using the FastDNA™ SPIN kit(MP Biomedicals, Eschwege, Germany) according to the manufacturer’s instructions. The quality and concentration of DNA samples were determined by 1.0% agarose gel electrophoresis and a NanoDrop^®^ ND-2000 spectrophotometer (Thermo Scientific Inc., USA) and they were kept at −80 °C for further use. The hypervariable region V5–V7 of the bacterial 16S rRNA gene was amplified with the primer pairs 799F (5′-AACMGGATTAGATACCCKG-3′) and 1193R (5′-ACGTCATCCCCACCTTCC-3′) [28] using an ABI GeneAmp 9700 PCR thermocycler (ABI Applied Biosystems, Foster City, CA, USA). The PCR mixture included 4 μL 5 × Fast Pfu buffer, 2 μL 2.5 mM dNTPs, 0.8 μL of each primer (5 μM), 0.4 μL Fast Pfu polymerase, 0.2 μL BSA, 10 ng of template DNA, and ddH_2_O to a final volume of 20 µL.

The PCR amplification cycling conditions were: initial denaturation at 95 °C for 3 min; the first round of amplification followed by 27 cycles of denaturation at 95 °C for 30 s, annealing at 55 °C for 30 s, extension at 72 °C for 45 s, and a single extension cycle at 72 °C for 10 min; the second round of amplification was for 13 cycles, with the amplification conditions remaining unchanged followed by 10 °C cooling.

The ITS1 region of the fungus was amplified with primers ITS1F (5′-CTTGGTCATTTAGAGGAAGTAA-3′) and ITS2R (5′-GCTGCGTTCTTCATCGATGC-3′). The PCR mixture included 2 μL 10× buffer, 2 μL 2.5 mM dNTPs, 0.8 μL of each primer (5 μM), 0.2 μL rTaq Polymerase, 0.2 μL BSA, 10 ng of template DNA, and ddH_2_O to a final volume of 20 µL. All samples were amplified in triplicate. The PCR product was extracted from 2% agarose gel, purified using the AxyPrep DNA Gel Extraction Kit (Axygen Biosciences, Union City, CA, USA) according to the manufacturer’s instructions and quantified using a Quantus™ Fluorometer (Promega, Madison, WI, USA). PCR amplification and Illumina MiSeq sequencing were performed by the Majorbio Bio-Pharm Technology Co. Ltd. (Shanghai, China) and the original data were uploaded to GenBank with project login number PRJNA1007442. Available at: https://www.ncbi.nlm.nih.gov/bioproject/PRJNA1007442/ (Accessed on 21 August 2023).

### 2.3. Environmental Factor Determination

The HOBO U30 compact automatic weather station was utilized for long-term monitoring of meteorological factors within the experimental area. The environmental factors monitored include air relative humidity, effective solar radiation, air temperature, soil temperature, and precipitation. Measurements were taken at 1 h intervals. We calculated the average values for air relative humidity, effective solar radiation, air temperature, and soil temperature over a season to obtain mean seasonal humidity, mean seasonal solar radiation, mean seasonal air temperature, and mean seasonal soil temperature values. The sum of the precipitation over a season was computed to determine the mean seasonal precipitation. The meteorological data were then organized to present the environmental factor information for the sampling points as shown in Table 2.

### 2.4. Statistical Analysis

Bioinformatics analysis was performed using the Majorbio Cloud Platform (https://cloud.majorbio.com, accessed on 21 August 2023.). Flash software (version 1.2.11) was used to splice the original sequencing data and Trimmomatic software (version 0.33) was used to filter the spliced data. The operational taxonomic units (OTUs) were classified based on a 97% sequence similarity level using Usearch software (version 11) after annotation and comparison with the Silva (https://www.arb-silva.de/ accessed on 21 August 2023) and Unite (Release 8.0, https://unite.ut.ee/ accessed on 21 August 2023) databases. Alpha-diversity indices were calculated using Mothur software (version 1.30.2) and *t*-tests were performed using R software (version 3.5.1) to analyze the differences in alpha-diversity indices between the groups. The beta-diversity distance matrix was calculated based on Qiime software (version 1.9.1) and the results of ANOSIM inter-group difference test were integrated. The sampling R language (vegan software package) was used for NMDS non-metric multidimensional scaling analysis of the samples. LEfSe multilevel-species-difference discriminant analysis was used to evaluate the flora that had a significant influence on sample differences and linear discriminant analysis (LDA) was used to screen out the flora that had an important influence on the differences between groups (LDA score > 2) [29]. An RDA model was used for the correlation analysis of environmental factors (R language version 2.4.3). Functional prediction analysis of endophytic bacteria was performed using the FAPROTAX database, and COG family information corresponding to the endophytic bacterial OTUs was obtained through comparison. The FUNGuild predictive tool was used to classify each endophytic fungal community into eight functional nutritional types, including pathotrophs (PA), symbiotrophs (SY), saprotrophs (SA), their combinations, and unknown types (UN). Significant differences in bacterial functional groups were determined using the Wilcoxon rank-sum test for inter-group analyses.

## 3. Results

### 3.1. Diversity Analysis of Endophytic Bacteria in Actinidia arguta

The sequencing yielded 1,251,952 valid endophytic bacterial sequences annotated to 2708 OTUs and 1,552,898 valid endophytic fungal sequences annotated to 588 OTUs; the average sequencing coverage reached 99.88% and 99.96%, respectively.

In the alpha-diversity analysis (Table 3 and Table 4), the Chao and Shannon indices of endophytic bacteria indicated that bacterial community richness was highest in winter and lowest in summer, with statistically significant differences between seasonal bacterial richness (*p* = 0.005, *p* = 0.008). Comparison of the Chao index of endophytic fungi across the four sample groups showed the highest value in summer, followed by winter, and the lowest in spring. Statistical analysis revealed a significant difference in endophytic fungal richness across different seasons (*p* = 0.0009). Conversely, the Shannon index of endophytic fungal communities was highest in winter, followed by summer, and lowest in autumn, with no statistically significant differences in endophytic fungal diversity between different seasons (*p* = 0.436).

**Figure 1:**
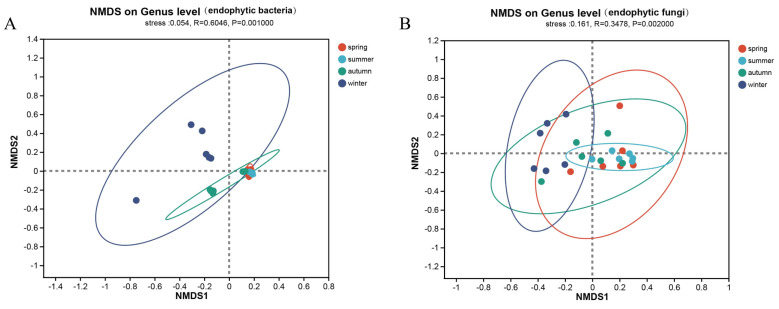
Beta−diversity analysis of endophytes in *Actinidia argute.* (**A**) The NMDS analysis based on the ANOSIM inter-group difference test in the beta-diversity analysis of the endophytic bacterial flora; (**B**) The NMDS analysis based on the ANOSIM inter-group difference test in the beta-diversity analysis of the endophytic fungal flora.

The NMDS analysis based on the ANOSIM inter-group difference test in the beta-diversity analyses (Figure 1A,B) showed significant differences in the structure of the endophytic bacterial flora (R = 0.605, *p* = 0.001) and endophytic fungal communities (R = 0.348, *p* = 0.002) between seasons and the groupings explained some of the differences in the structure of the endophytic bacterial and fungal flora (stress = 0.054, stress = 0.161).

### 3.2. Community Composition and Diversity Analysis of Actinidia arguta Endophytes

**Figure 2:**
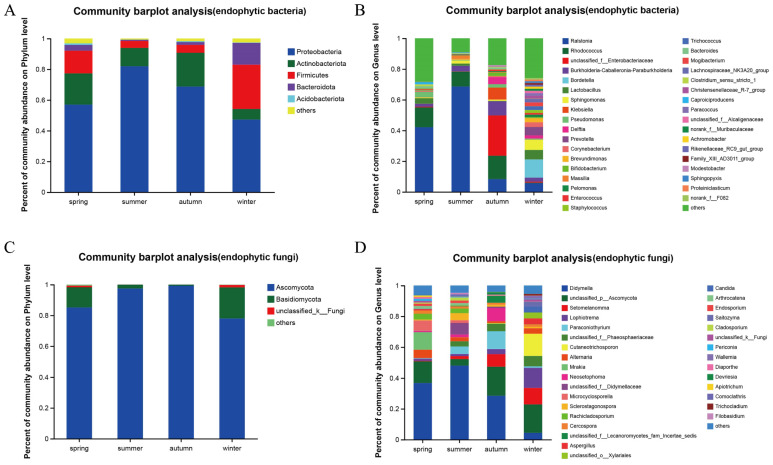
Microbial community-structure analysis of endophytes based on genus and phylum levels. (**A**) Percent of community abundance at phylum level of endophytic bacteria. (**B**) Percent of community abundance at genus level of endophytic bacteria. (**C**) Percent of community abundance on phylum level of endophytic fungi. (**D**) Percent of community abundance at genus level of endophytic fungi.

For this analysis, 2708 endophytic bacterial OTUs were compared with 36 phyla and 897 genera. At the phylum classification level (Figure 2A), *Proteobacteria* were predominant in the spring, summer, autumn and winter samples (56.98%, 81.95%, 68.75%, 47.22%); Actinobacteriota were subdominant in the spring, summer, and autumn samples (20.33%, 11.93%, 21.97%), the Firmicutes had a secondary dominance in winter and spring (28.75%, 14.78%). The proportion of the communities composed by Bacteroidota in winter is also relatively high (14.21%), and the proportion of Acidobacteriota in the four seasons is no more than 1.07%.

At the generic level, the spring samples contained 481 bacterial genera (Figure 2B) and *Ralstonia* (42.11%), *Rhodococcus* (12.86%), and *Lactobacillus* (3.60%) had a relative abundance greater than 3% and the relative abundance for the other genera was greater than 28.34%. The summer sample had 315 genera, including *Ralstonia* (68.60%), *Rhodococcus* (9.72%), *Burkholderia-Caballeronia-Paraburkholderia* groups (3.60%), and there were three genera with relative abundance greater than 3% and 9.14% for the other genera. The autumn samples contained 450 genera, with Unclassified_f_Enterobacteriaceae group (26.25%), *Rhodococcus* (15.20%) and *Burkholderia-Caballeronia-Paraburkholderia* group (9.18%), there were six genera with relative abundance greater than 3% and 17.14% for the other genera. The relative abundance of the 562 genera in the winter sample was greater than 3% for *Bordetella* (11.85%), *Sphingomonas* (6.65%), and *Lactobacillus* (6.15%) genera and 25.89% for the other genera. The total number of genera in the spring, summer, autumn and winter samples was 157, accounting for 17.50% of all species of endophytic bacteria and 90.21% of the total abundance of the community.

A total of 588 endophytic fungal OTUs were compared to eight phyla and 251 genera. At the phylum level (Figure 2C), Ascomycota was a major dominant phylum in stem tissues during spring, summer, autumn, and winter (85.14%, 97.46%, 99.26%, and 77.99%) and its relative abundance was higher in summer and autumn samples than in spring and winter samples. Basidiomycota was more abundant in winter and spring (20.20% and 13.09%) than in summer and autumn (2.50% and 0.73%). Unclassified_k_Fungi and unknown fungi were found in small amounts in spring and winter samples (1.07% and 0.70%, 1.58% and 0.22%, respectively), whereas no unknown fungi were found in summer samples.

At the genus level (Figure 2D), the spring, summer, autumn and winter samples had 84, 133, 83, and 140 genera, respectively. In the spring samples, six genera, including *Didymella* (36.79%), Unclassified_p_Ascomycota (14.04%), and *Mrakia* (11.43%), had a relative abundance greater than 3%, whereas that of the others was 6.44%. In the summer samples, seven genera, including *Didymella* (48.07%), Unclassified_f_Didymellaceae (14.04%), and *Paraconiothyrium* (4.86%), had a relative abundance greater than 3%, whereas that of the others was 4.71%. In the autumn samples, seven genera, including *Didymella* (28.50%), Unclassified_p_Ascomycota (18.86%), and *Paraconiothyrium* (11.58%), had a relative abundance greater than 3%, whereas that of the others was 4.19%. In the winter samples, ten genera, including Unclassified_p_Ascomycota (18.44%), *Cutaneotrichosporon* (14.45%), and *Lophiotrema* (13.10%), had a relative abundance greater than 3%, whereas that of the others was 5.43%. The spring, summer, autumn, and winter samples had 32 bacterial genera in common, accounting for 12.75% of all species of endophytic bacteria and 86.54% of the total abundance of the flora.

**Figure 3:**
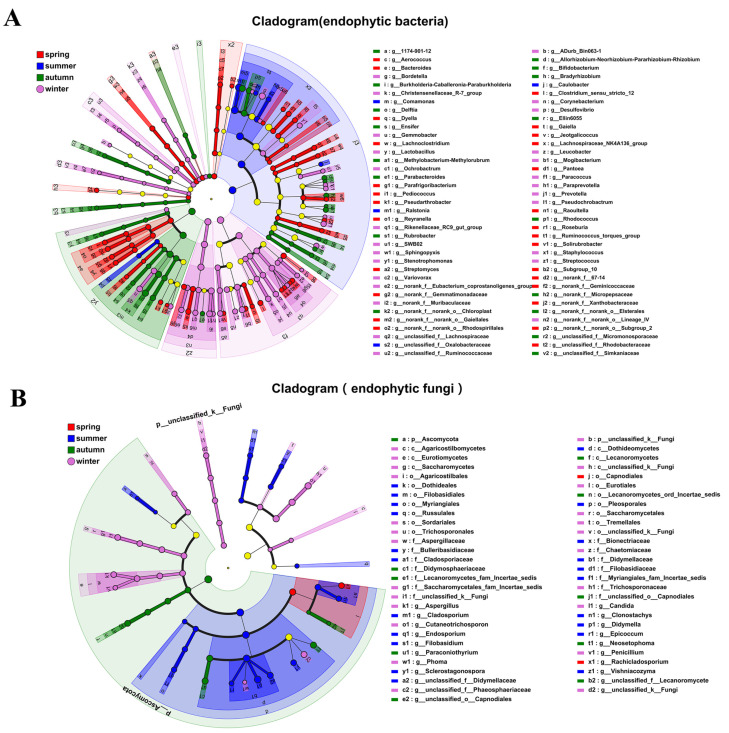
LEfSe multilevel phylogenetic tree diagram of endophytic fungi in *Actinidia argute*. (**A**,**B**) LEfSe multilevel-species hierarchical analysis of endophytic bacteria and fungi. From the inner to the outer circle of the LEfSe multilevel-species hierarchy tree represents the classification level of the flora from phylum to genus. The diameter of node circles is proportional to the relative abundance. The yellow nodes represent the flora with no significant differences between samples, while the red, blue, green, and pink nodes represent the flora with significant differences between samples. Red nodes indicate the flora with a comparative advantage in spring stem tissues, blue nodes indicate the flora with a comparative advantage in summer stem tissues, green nodes indicate the flora with a comparative advantage in autumn stem tissues, and pink nodes indicate the flora with a comparative advantage in winter stem tissues.

LEfSe multilevel-species hierarchical analysis showed that at the phylum level of endophytic bacteria Chloroflexi, Acidobacteriota, and Fusobacteriota were relatively dominant in spring; *Proteobacteria* were relatively dominant in summer; Actinobacteriota and Nitrospirota were relatively dominant in autumn; and Firmicutes, Bacteroidota, and Spirochaetota were relatively dominant in winter (Figure 3A). At the phylum level of endophytic fungi, there were no dominant phyla in spring or summer, *Ascomycota* was relatively dominant in autumn, and *Basidiomycota* and Unclassified_k_Fungi were relatively dominant in winter. At the genus level, 13, 3, 7, and 12 genera were relatively dominant in spring, summer, autumn and winter, respectively. Among the endophytic fungi, 1, 9, 3, and 5 genera were relatively dominant in spring, summer, autumn, and winter, respectively (Figure 3B).

**Figure 4:**
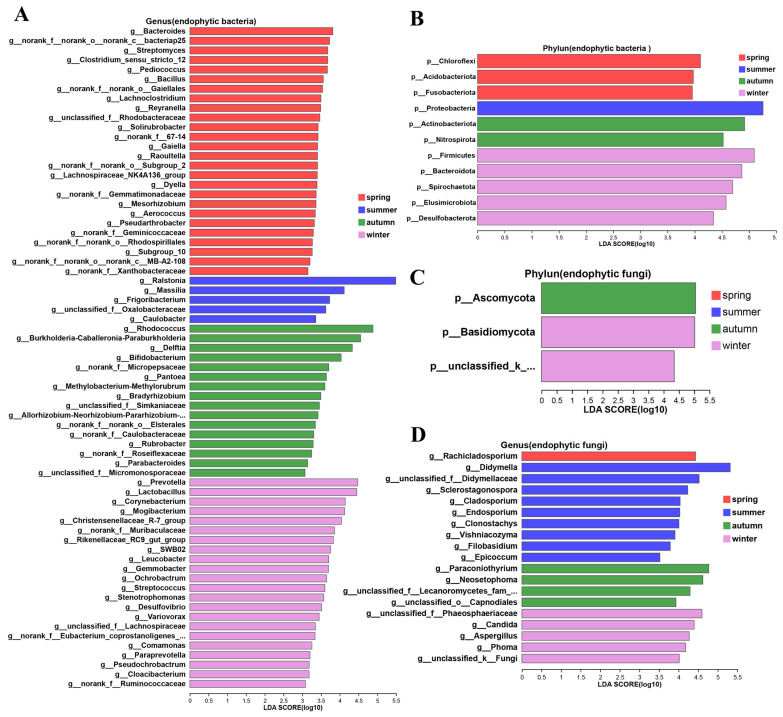
LDA discriminant analysis of endophytic fungi in *Actinidia argute*. (**A**,**B**) Linear discriminant analysis of endophytic bacteria. (**C**,**D**) Linear discriminant analysis of endophytic fungi.

LDA showed that 69 genera of endophytic bacteria, including *Bacteroides*, *Ralstonia*, *Rhodococcus*, and *Prevotella*, significantly affected the differences between the seasons. There were 26, 5, 16, and 22 differentially abundant genera in the spring, summer, autumn, and winter samples, respectively. These bacterial genera perform various functions in plants. For example, *Frigoribacterium* [30] and *Pseudochrobactrum* [31] have cold-tolerant functions, *Variovorax* [32] has growth-promoting functions, *Streptomyces* [33] has disease-resistance functions, and *Desulfovibrio* [34] and *Stenotrophomonas* [35] are able to degrade heavy metals and pesticide pollution (Figure 4A,B). Among the endophytic fungi, 19 genera such as *Rachicladosporium*, *Didymella*, *Paraconiothyrium*, and *Candida* had significant influences on the differences between different seasons. These fungi have different functions in plants. For example, *Cladosporium*, [36] *Clonostachys*, and *Paraconiothyrium* have antibacterial activity [37] (Figure 4C,D).

### 3.3. Effects of Environmental Factors on the Community Structure of Endophytic Bacteria in Actinidia arguta

**Figure 5:**
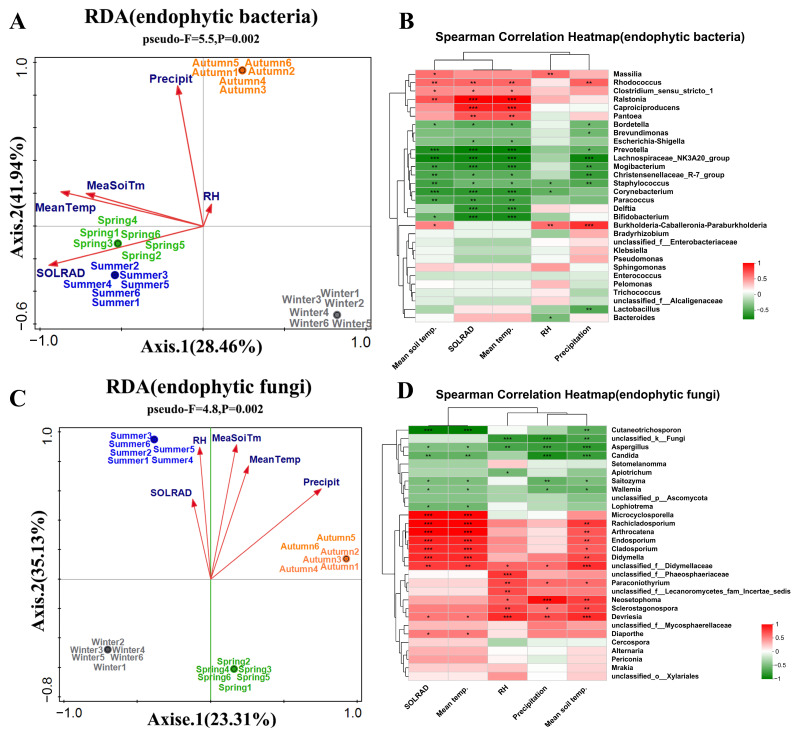
Effect of environmental factors on the microbial community structure of endophytes in *Actinidia arguta.* (**A**,**B**) Redundancy analysis (RDA) was employed to examine the interrelationships among environmental factors, samples, and microbial communities. (**C**,**D**) Correlation Heatmap was utilized to analyze the correlations between environmental factors and selected species. The color bar in the legend represents data values through varying shades, with red indicating positive correlations and green indicating negative correlations. Note: In the figure, MeaSoiTm stands for soil temperature; SOLRAD stands for solar radiation; Mean Temp stands for temperature; RH stands for relative air humidity; and Precipit stands for quarterly precipitation; * means significant difference between samples (*p* < 0.05); ** means significant difference between samples (*p* ≤ 0.01); *** means significant difference between samples (*p* ≤ 0.001).

For endophytic bacteria, environmental factors exhibited a significant positive correlation with the community structure during spring, summer, and autumn, whereas a negative correlation was observed in winter (Figure 5A). The principal influencing factors included SOLRAD, Mean Temp, and Precipit. Specifically, MeaSoiTm and Mean Temp significantly and positively correlated with the abundance of eight bacterial genera, including *Ralstonia*, *Rhodococcus*, and *Massilia*, while precipitation markedly influenced the abundance of *Rhodococcus* and *Burkholderia-Caballeronia-Paraburkholderia* (Figure 5B).

Regarding endophytic fungi, environmental factors showed a positive correlation with the fungal community structure in summer and autumn, but this correlation was reversed in spring and winter (Figure 5C). The key influencing factors comprised RH, MeaSoiTm, Mean Temp, and Precipit. Genera such as *Microcyclosporella*, *Rachicladosporium*, and *Arthrocatena* were significantly positively correlated with mean temperature. Some genera, including *Neosetophoma*, *Sclerostagonospora*, and *Devriesia* were significantly influenced by RH and Precipit. *Endosporium*, *Cladosporium*, *Didymella*, among others, were significantly affected by MeaSoiTm (Figure 5D). These findings suggest that environmental factors such as MeaSoiTm, Mean Temp, Precipit, and SOLRAD significantly impact the community structure of endophytic bacteria and fungi across different seasons, with certain genera displaying clear positive correlations with specific environmental conditions.

### 3.4. Analysis of Functional Composition and Inter-Group Differences of Endophytic Bacteria in Actinidia arguta

**Figure 6:**
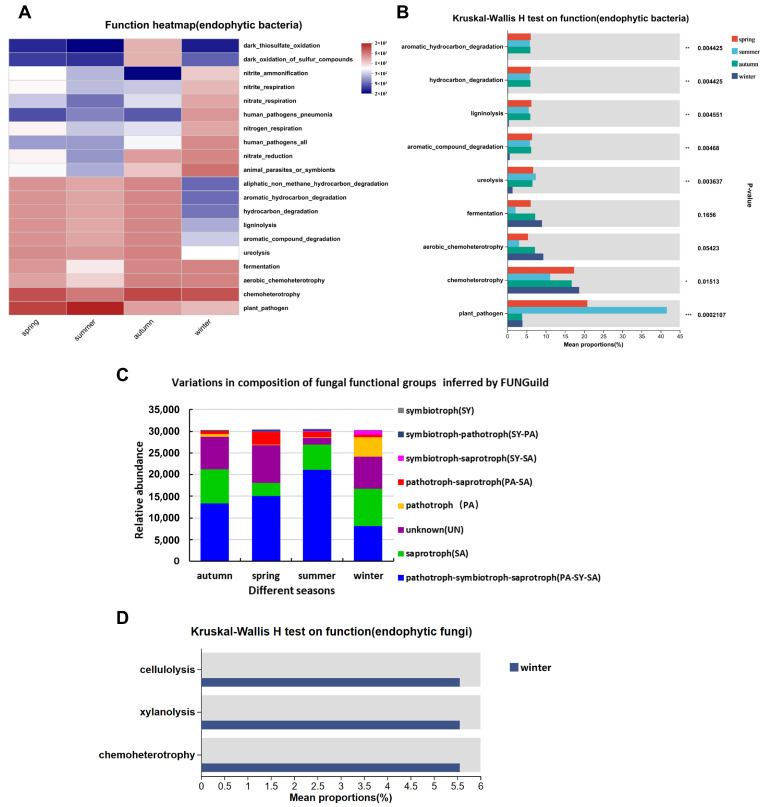
Analysis of functional composition and inter-group differences of endophytic bacteria in *Actinidia argute*. (**A**) Inter-group analysis of FAPROTAX-function composition of endophytic bacteria; (**B**) Kruskal–Wallis H test on function of endophytic bacteria; (**C**) variations in composition of fungal functional groups inferred by FUNGuild; and (**D**) Kruskal–Wallis H test on function of endophytic fungi. Note: In the figure, PA represents pathotroph; SY represents symbiotroph; SA represents saprotroph; and UN represents unknown; * means significant difference between samples (*p* < 0.05); ** means significant difference between samples (*p* ≤ 0.01); *** means significant difference between samples (*p* ≤ 0.001).

Utilizing FAPROTAX and FUNGuild, this study predicts the functional capabilities of endophytes in *Actinidia arguta*, exploring the potential interactions between microbial communities and the host plant. Functional analysis using the FAPROTAX database for the top 20 abundant endophytic bacterial genera revealed chemoheterotrophy, *aerobic* chemoheterotrophy, fermentation, and ureolysis as the most abundant functions. The functional similarity between spring and summer contrasted with that between autumn and winter, particularly for functions like aromatic-hydrocarbon degradation and ligninolysis, which were significantly lower in winter (*p* < 0.01) (Figure 6A). Conversely, fermentation and aerobic chemoheterotrophy were higher in winter but with no significant difference (*p* = 0.1656, *p* = 0.0542). Chemoheterotrophy was significantly higher in winter (*p* < 0.05), while plant pathogens were more abundant in summer than in spring, significantly so compared to autumn and winter (*p* < 0.01) (Figure 6B).

FUNGuild analysis showed significant differences in the nutritional patterns of endophytic fungi in different seasons, with PA-SY-SA (44.26%) as the main factor in spring (Figure 6C), followed by SA (25.96%) and UN (24.49%). In summer, PA-SY-SA (49.60%) was dominant, followed by UN (28.55%) and SA (10.19%). In autumn, PA-SY-SA (69.63%) was dominant, followed by SA (18.76%) and UN (5.40%). In winter, SA (28.44%) was dominant, followed by PA-SY-SA (27.07%) and UN (24.37%). The results of the survey of the common functions in the group are shown in Figure 6D, and there was no difference in the endogenous fungal function of the spring, summer, and autumn samples, whereas the functions of cellulolysis, xylanolysis, and chemoheterotrophy in winter were significantly different from the other seasons.

Analysis of the top 15 most abundant endophytic bacterial genera in *Actinidia arguta* (Table 5) revealed 10 genera belonging to *Proteobacteria* and 2 to *Actinobacteria*, with 1 each from *Firmicutes*, *Bacteroidetes*, and *Acidobacteria*. *Ralstonia*, dominant in spring and summer, saw a gradual increase in abundance, peaking in summer and declining towards winter, with a winter abundance of only 5.71%, indicating its importance in the microecosystem of *Actinidia arguta*. *Rhodococcus* was the second most dominant genus during spring and summer, displaying sensitivity to winter’s low temperatures and contributing to the ecological balance in warmer seasons. The Unclassified_f_Enterobacteriaceae genus dominated in autumn, but was suppressed in other seasons and sensitive to cold. *Burkholderia-Caballeronia-Paraburkholderia* detection was distributed across seasons, peaking in autumn. *Bordetella* detection was exclusive to winter, suggesting a specific role during the cold. *Sphingomonas* and *Lactobacillus* also showed higher winter abundance. *Klebsiella*, present only in spring and autumn, is considered cold-sensitive and suppressed by dominant genera in other seasons, aiding plant growth when nutrients are scarcer in autumn. *Delftia*, found only in autumn and winter, may help in stress resistance, while *Prevotella*, only present in winter, is cold-tolerant and possibly active during plant dormancy. *Corynebacterium*, absent in summer but abundant in winter, may play a role in osmotic stress resistance. *Massilia*, present in summer and autumn, is likely cold-sensitive.

Analysis of the ten most abundant endophytic fungal genera in *Actinidia arguta* (Table 6) shows that eight genera are from the phylum *Ascomycota* and two are from *Basidiomycota*. *Didymella* emerges as the predominant genus in spring and summer, maintaining the ecological balance of *Actinidia arguta* with growth-promoting functions. The *unclassified_p_Ascomycota* genus, dominant in winter and the second most prevalent in spring and autumn, is suppressed by *Didymella* in summer. *Lophiotrema* and *Setomelanomma*, increasing from spring to winter, may enhance plant stress resistance. *Paraconiothyrium*, detected from summer to winter with its highest content in autumn, is suggested to be cold-sensitive. *Cutaneotrichosporon*, found only in winter, is a cold-tolerant genus secreting lipids. *Alternaria* shows a rise and fall from winter to autumn, with its highest abundance in spring, indicating involvement in carbohydrate metabolism in winter and nitrogen metabolism in spring. *Mrakia*, present only in spring as the secondary dominant strain, likely aids the plant in resisting low temperatures through the production of cold-active enzymes and secondary metabolites.

## 4. Discussion

The Illumina MiSeq technology allows not only comprehensive and objective ascertainment of the relative composition of microbial communities in target environments but also aids in the study of the diversity of symbiotic microbial ecological functions [26]. This technique has been widely applied to the study of gut microbiome diversity [96], soil microbial diversity [97], and space microbiology [98], among other study areas. To date, research utilizing this technology on the endophytes of the Actinidia genus has primarily focused on analyzing the internal microbial community compositions of various tissues like fruits, pollen, and the sap of different kiwifruit varieties [99]. However, the study of endophytic bacteria within *Actinidia arguta* using this technology remains unexplored.

This study employed Illumina MiSeq sequencing technology to analyze, for the first time, the community structure and species abundance of endophytic bacteria and fungi in *Actinidia arguta*. The sequencing coverage exceeded 99.8%, showcasing the richness and diversity of the endophytic microbial community within *Actinidia arguta*. The endophytic bacteria were identified across 36 phyla and 897 genera, with the dominant phyla being *Proteobacteria*, *Actinobacteriota*, *Firmicutes*, and *Bacteroidota*. Endophytic fungi were identified in eight phyla and 251 genera, predominantly within *Ascomycota* and *Basidiomycota*. Cho et al. found similar dominant bacterial phyla in the overflowing sap of different kiwifruit varieties, aligning with our findings [100]. Kim et al. identified the dominant bacterial phyla in the sap of the CV kiwifruit variety [99]. Deliwoong reported the dominant bacterial phyla as *Proteobacteria*, *Actinobacteria* and *Firmicutes*, and the dominant fungal phyla as *Ascomycota*, *Mortierellomycota*, and *Basidiomycota*, with one extra phylum compared to our study, possibly due to environmental microbial differences [101]. To date, *Actinidia arguta* has represented the species with the richest microbial community within the Actinidia genus. The study of the overflowing sap in three kiwifruit varieties, including Hayward, identified endophytic bacteria within only 12 phyla and 150 genera. This conclusion lays a foundation for the further exploration of endophytic microbial resources in *Actinidia arguta* [100].

In natural ecosystems, plants coexist in a state of mutual dependence with microorganisms [102]. The dynamics of plant endophytic microbial communities are influenced by intrinsic factors such as host species [103], genotype [104], organ [105], developmental stage [106], and nutritional status [105], as well as by external environmental factors, including soil type [107], fertilization practices [101], and seasonal changes [108]. This study focused on the impacts of seasonal variations on the structure of endophytic communities. Analysis of alpha (*p* < 0.01) and beta (*p* < 0.01) diversity revealed significant differences in the structure and diversity of endophytic communities in *Actinidia arguta* across different seasons, underscoring seasonal change as a key determinant [109].

In the realm of studies on endophytic microbial diversity, most researchers posit that the diversity of endophytes during winter tends to decrease due to factors such as low temperatures and reduced solar radiation [110]. However, the diversity of both endophytic bacteria and endophytic fungi in *Actinidia arguta* is paradoxically highest during winter. Genera enriched specifically during the winter season include *Bordetella*, *Prevotella*, and *Cutaneotrichosporon*. Studies have shown that dophytes from Eucommia ulmoides bark [17] and Camellia yuhsienensis Hu roots [111] also displayed higher diversities in winter compared to other seasons. We hypothesize that because *Actinidia arguta* is highly cold-resistant, the endophytic microbes have gradually adapted to the cold winter conditions during their co-evolution with this plant. Furthermore, these endophytes likely play a significant role in assisting *Actinidia arguta* in coping with abiotic stress factors.

In terms of community structure, the differences between seasons are more pronounced [112]. A series of studies indicates that changes in climate factors such as solar radiation, temperature, rainfall, and humidity due to seasonal variations are crucial influencers of microbial communities [113]. Our research further substantiates this point; we have observed distinct differences in the community structure of endophytic microbes within Actinidia arguta across different seasons. These differences are influenced to varying degrees by solar radiation, temperature, soil temperature, rainfall, and air humidity. Moreover, during winter, the endophytic microbial community shows a negative correlation with these environmental factors. Analysis of the top thirty most abundant endophytic bacteria and fungi indicated that 66.67% of bacterial and 32% of fungal dominant genera are significantly influenced by environmental factors, such as temperature affecting genera like *Ralstonia*, *Rhodococcus*, and *Massilia*, while precipitation significantly impacts genera like *Neosetophoma*, *Sclerostagonospora*, and *Devriesia*. This further corroborates our previous findings that endophytic bacterial richness and diversity are greater during winter when solar radiation is weaker and temperatures are lower, demonstrating the strong environmental stress tolerance of endophytes in *Actinidia arguta*.

Endophytes maintain their growth by absorbing nutrients from plants, yet they also perform various functions that contribute to plant growth [4,5]. Utilizing FAPROTAX and FUNGuild to predict the functions of endophytes, it was found that the endophytic bacteria of *Actinidia arguta* primarily engage in functions such as chemoheterotrophy, oxidation, nitrate reduction, respiration, denitrification, and degradation, with significant seasonal variations. The nutritional modes of endophytic fungi in spring, summer, and autumn are mainly PA-SY-SA, while in winter, SA prevails. There are significant functional differences between the endophytic fungi of spring, summer, and autumn compared to winter, with winter exhibiting pronounced abilities in cellulolysis, xylanolysis, and chemoheterotrophy.

The primary functions of endophytes are directly related to the abundance of dominant genera across different seasons [5]. *Ralstonia* and *Rhodococcus* are the primary and secondary dominant genera in spring and summer, respectively, shaping the microbial structure of *Actinidia arguta* during these seasons [40] and potentially involved in biodegradation [41], bioremediation, and biotransformation [7]. The genus Unclassified_f_Enterobacteriaceae, dominant in autumn, is suppressed by *Ralstonia* and *Rhodococcus* and is sensitive to low temperatures. This genus is presumed to facilitate plant nutrient recirculation through chemoheterotrophy, ligninolysis, and fermentation, thus preparing the plant for winter [44]. *Bordetella*, found exclusively in winter and dominant during this season, is hypothesized to assist *Actinidia arguta* in coping with low temperatures by expressing antifreeze proteins [50]. *Klebsiella*, present in spring and autumn with a peak abundance of 7.72% in autumn, is considered cold-sensitive and is suppressed by dominant genera in other seasons, while aiding plant growth and enhancing disease resistance during the nutrient-scarce autumn [9]. *Prevotella*, exclusive to winter, is speculated to be cold-tolerant and active during plant dormancy, is severely suppressed by dominant genera in other seasons, and is thought to aid in plant adaptation to harsh winter climates through the degradation of proteins, sugars, and lignin [63,64]. *Corynebacterium*, absent in summer but abundant in winter, is believed to be suppressed by other genera in summer and helps the plant to withstand freezing stress in winter through osmotic stress tolerance [67].

*Didymella* is the dominant endophytic fungal genus in *Actinidia arguta* during spring, summer, and autumn, with its highest abundance in summer, suggesting that its primary role lies in promoting plant growth and aiding disease resistance [81]. The genus Unclassified_p_Ascomycota, prevalent in winter and the second most dominant genus in spring and autumn, exhibits low summer abundance, potentially due to suppression by *Didymella*, and is hypothesized to enhance plant stress resistance through the secretion of active secondary metabolites [83]. *Cutaneotrichosporon*, present only in winter as the second most dominant genus, is analyzed to be cold-tolerant, possibly secreting lipids to alter cellular fluid composition and enhance plant frost resistance. *Mrakia*, exclusive to spring and the third most dominant strain, is presumed to play a significant role in assisting the plant to withstand late cold spells during the pre-sprouting phase in spring [12,114].

Analyzing the endophytic microbial community structure and diversity of *Actinidia arguta* from a seasonal perspective not only enhances our understanding of its microecosystem and potential pathogenic microbes but also provides references for mining functional microbial strains within the plant [115]. This allows for the determination of optimal sampling seasons and the advance verification of culture conditions for targeted microbial genera, thereby increasing the likelihood of successful functional strain isolation [116].

## 5. Conclusions

Illumina MiSeq sequencing revealed a rich diversity of endophytic bacteria and fungi within *Actinidia arguta*, with OTUs belonging to 36 phyla and 897 genera and 8 phyla and 251 genera, respectively. Notable seasonal shifts were observed: *Proteobacteria* and *Actinobacteriota* dominated from spring to autumn, whereas *Proteobacteria*, *Firmicutes*, and *Bacteroidota* were prevalent in winter. In addition, season-specific genera such as *Mrakia* were identified in spring and *Bordetella* and *Prevotella* in winter. Temperature and rainfall significantly influenced microbial community structure. The functional roles of endophytes also varied with the seasons, particularly in winter, where endomicrobes demonstrated unique functions in cellulolysis and ureolysis, contributing to the growth and environmental stress resilience of *Actinidia arguta*.

## Figures and Tables

**Table 1:** Information ofabout sampling time and environmental factors.

Sampling Season	Sampling Date (Y.M.D)	RH (%)	Precipit (mm)	SOLRAD (W/m^2^)	Mean Temp (°C)	MeaSoiTm (°C)
Winter	15 January 2021	73.66	13.60	80.05	−16.09	−4.54
Spring	15 April 2021	56.32	21.30	183.60	6.50	4.45
Summer	15 July 2021	78.43	24.20	245.01	23.38	24.50
Autumn	15 October 2021	66.69	22.70	127.78	6.32	9.92

Note: In the table, MeaSoiTm stands for soil temperature; SOLRAD stands for solar radiation; Mean Temp stands for temperature; RH stands for relative air humidity; and Precipit stands for quarterly precipitation.

**Table 2:** Environmental information for the sample-collection points.

Sampling Season	RH (%)	Precipit (mm)	SOLRAD (W/m^2^)	Mean Temp (°C)	MeaSoiTm (°C)
Spring	63.75	59.20	177.24	7.01	5.77
Summer	80.77	179.00	213.81	21.82	22.61
Autumn	75.99	299.10	109.38	6.39	10.53
Winter	72.24	19.90	88.99	−13.27	−2.92

Note: In the table, MeaSoiTm stands for soil temperature; SOLRAD stands for solar radiation; Mean Temp stands for temperature; RH stands for relative air humidity; and Precipit stands for quarterly precipitation.

**Table 3:** Alpha-diversity-index analysis of endophytic bacteria in *Actinidia arguta* based on the Student’s *t*-test.

Endophyte Type	Index Type	Spring Stem (n = 6)	Summer Stem (n = 6)	Autumn Stem (n = 6)	Winter Stem (n = 6)	*p* Value
Endophytic Bacteria	Coverage	0.9990 ± 0.0002	0.9990 ± 0.0002	0.9966 ± 0.0021	0.9984 ± 0.0006	0.0009 ***
	Chao	347.73 ± 155.68	232.73 ± 28.25	454.46 ± 197.44	467.86 ± 180.96	0.005 **
	Shannon	3.01 ± 0.71	1.7211 ± 0.30	2.81 ± 0.50	3.86 ± 1.23	0.008 **
Endophytic Fungi	Coverage	0.9999 ± 0.0000	0.9999 ± 0.0000	1.0000 ± 0.0000	0.9999 ± 0.0000	0.001 ***
	Chao	31.75 ± 6.31	68.74 ± 13.07	32.58 ± 4.68	56.94 ± 24.15	0.0009 ***
	Shannon	1.65 ± 0.40	1.95 ± 0.46	1.61 ± 0.5212	1.99 ± 0.40	0.436

Note: The values in the table represent the mean ± standard deviation; ** means significant difference between samples (*p* ≤ 0.01) and *** means significant difference between samples (*p* ≤ 0.001).

**Table 4:** Statistical differences between the endophytic microbial communities of Actinidia arguta across four seasons.

Endophyte Type	Index Type	*p* Value (Autumn–Spring)	*p* Value (Autumn–Summer)	*p* Value (Winter–Autumn)	*p* Value (Spring–Summer)	*p* Value (Spring–Winter)	*p* Value (Summer–Winter)
Endophytic Bacteria	Coverage	<0.01	<0.01	<0.1	≥0.1	≥0.1	≥0.1
	Chao	<0.05	<0.01	≥0.1	≥0.1	≥0.1	<0.1
	Shannon	≥0.1	≥0.1	≥0.1	<0.1	≥0.1	<0.01
Endophytic Fungi	Coverage	≥0.1	<0.01	≥0.1	<0.01	≥0.1	≥0.1
	Chao	≥0.1	<0.001	<0.1	<0.001	≥0.1	<0.1
	Shannon	≥0.1	≥0.1	≥0.1	≥0.1	≥0.1	≥0.1

**Table 5:** Community structure and functional analysis of the top 15 most abundant endophytic bacterial genera in *Actinidia arguta*.

Endophytic Bacterial Genus	Phylum	Spring (%)	Summer (%)	Autumn (%)	Winter (%)	Functions
*Ralstonia*	Proteobacteria	42.11	66.68	8.35	5.71	Some species within this genus are potential pathogens [38]; some possess unique protein synthesis pathways to adapt to specific lifestyles [39]; in some plants, an anomalous increase in *Ralstonia* may alter the overall ecosystem of the microbial community [40].
*Rhodococcus*	Actinobacteria	12.76	9.61	15.18	0.34	Biodegradation [41]; biosurfactants and bioflocculants production [42]; indicators in hydrocarbon deposits for crude oil exploration; enhancing the aroma of noni juice [40]; adaptation to harsh environments and tolerance to toxic substances [43]; capabilities in biormediation and biotransformation [7].
Unclassified_f_Enterobacteriaceae	Proteobacteria	0.45	0.23	26.16	0.62	Genera related to plant defense [44].
*Burkholderia-Caballeronia-Paraburkholderia*	Proteobacteria	1.99	3.58	9.27	2.64	Certain species within the genus are potential plant pathogens [45]; some produce siderophores [46]; solubilize phosphates [47]; part of the ginsenoside-enriching microbial community [48].
*Bordetella*	Proteobacteria				11.89	Suppressing pathogens and enhancing plant disease resistance [49]; a genus characterized by high protein expression [50]; potential for degrading fertilizers and pesticides [51].
*Sphingomonas*	Proteobacteria	0.48	1.95	0.82	6.72	Involved in carbon, nitrogen, and sugar metabolism [52]; capable of synthesizing secondary metabolites such as welan gum and carotenoids to enhance plant stress resistance [53]; producing beneficial phytohormones [54].
*Klebsiella*	Proteobacteria	0.21		7.72		Promoting plant growth; biocontrol; degrading tebuconazole-class pesticides [9]; tolerance to the heavy metal Cd [55]; resilience to salinity and high temperatures [11].
*Pseudomonas*	Proteobacteria	3.59	0.34	2.15	0.66	Degradation of polycyclic aromatic hydrocarbons like phenanthrene and chloroacetamide herbicides [56]; phosphate solubilization and production of IAA for growth promotion [57]; remediation of mercury contamination [58]; disease resistance [59].
*Delftia*	Proteobacteria			4.61	1.98	Remediation of Cd contamination [60]; promotion of plant growth [61].
*Prevotella*	Bacteroidetes				5.51	Possessing the capability to degrade proteins and peptides [62]; xylanolytic activity [63]; promoting growth [64]; hemicellulose decomposition [65].
*Corynebacterium*	Acidobacteria	0.55		0.52	3.02	Antagonizing pathogenic fungi [66]; tolerance to salinity and osmotic stress [67]; growth promotion [68].
*Brevundimonas*	Proteobacteria		0.54	0.31	2.67	Production of indoleacetic acid (IAA), nitrogen fixation, phosphate solubilization, and siderophore production for growth promotion [69]; heavy metal tolerance [70].
*Massilia*	Proteobacteria		2.38	0.41		Soil fumigants [71]; degradation of polycyclic aromatic hydrocarbons like phenanthrene and chloroacetamide herbicides [72]; heavy metal resistance [73]; phosphate solubilization [74]; chitinase production [75]; production of cold-active enzymes such as mannanase, amylase, cellulase, and other polysaccharide hydrolases [76]; production of secondary metabolites like violacein [77] and polyhydroxyalkanoates [78].
*Bifidobacterium*	Actinobacteria			2.08	0.66	Probiotics commonly found in animal guts are also present on plants, including Actinidia arguta, with their functions yet to be determined [79].
**Total proportion**		65.22	84.55	76.56	37.36	

**Table 6:** Community structure and functional analysis of the top 10 most abundant endophytic fungal genera in *Actinidia arguta*.

Endophytic Fungi Genera	Phylum	Spring (%)	Summer (%)	Autumn (%)	Winter (%)	Function
*Didymella*	Ascomycota	36.76	47.96	28.55	4.41	Can survive in the soil for more than six months without a host [80]; possesses antibacterial activity [81] and promotes growth [82].
Unclassified_p_Ascomycota	Ascomycota	14.06	4.31	18.85	18.39	Affects the production of plant secondary metabolites such as saponins and flavonoids [83].
*Setomelanomma*	Ascomycota	0.26	1.81	8.04	10.80	Possesses biocontrol properties [84].
*Lophiotrema*	Ascomycota	1.35	1.41	3.32	13.07	Exhibits strong antibacterial effects [85].
*Paraconiothyrium*	Ascomycota		4.88	11.48	0.93	Has a wide range of hosts [37]; enhances plant drought resistance [86]; produces sesquiterpenoid compounds [87]; possesses biocontrol properties [88]; synthesizes titanium dioxide nanoparticles to reduce antibiotic use and lower the risk of antimicrobial resistance [89].
Unclassified_f__Phaeosphaeriaceae	Ascomycota		3.43	4.96	6.66	Unknown.
*Cutaneotrichosporon*	Basidiomycota				14.42	Produces oleaginous yeasts [90].
*Alternaria*	Ascomycota	5.39	1.79	1.21	3.55	Produces antimicrobial multicopper compounds [91]; generates nitrogen metabolites, steroids, terpenes, pyrones, quinones, and phenolic compounds [92]; promotes growth [93]; produces substances with antimicrobial activity [13].
*Mrakia*	Basidiomycota	11.43				Some species are cold-tolerant yeasts that ferment various sugars at low temperatures in response to the cold [94]; some produce gelatinase [95].
*Neosetophoma*	Ascomycota	0.75	1.79	8.52		Unknown.
Total proportion		70.00	67.38	84.93	72.23	

## Data Availability

The data presented in this study are available on request from the corresponding author.
